# These Squatters Are Not Innocent: The Evidence of Parasitism in Sponge-Inhabiting Shrimps

**DOI:** 10.1371/journal.pone.0021987

**Published:** 2011-07-21

**Authors:** Zdeněk Ďuriš, Ivona Horká, Petr Jan Juračka, Adam Petrusek, Floyd Sandford

**Affiliations:** 1 Department of Biology and Ecology, Faculty of Science, University of Ostrava, Ostrava, Czech Republic; 2 Department of Ecology, Faculty of Science, Charles University in Prague, Prague, Czech Republic; 3 Cedar Rapids, Iowa, United States of America; Technion-Israel Institute of Technology Haifa 32000 Israel, Israel

## Abstract

Marine sponges are frequently inhabited by a wide range of associated invertebrates, including caridean shrimps. Symbiotic shrimps are often considered to be commensals; however, in most cases, the relationship with sponge hosts remains unclear. Here we demonstrate that sponge-inhabiting shrimps are often parasites adapted to consumption of sponge tissues. First, we provide detailed examination of morphology and stomach contents of *Typton carneus* (Decapoda: Palaemonidae: Pontoniinae), a West Atlantic tropical shrimp living in fire sponges of the genus *Tedania*. Remarkable shear-like claws of *T. carneus* show evidence of intensive shearing, likely the result of crushing siliceous sponge spicules. Examination of stomach contents revealed that the host sponge tissue is a major source of food for *T. carneus*. A parasitic mode of life is also reflected in adaptations of mouth appendages, in the reproduction strategy, and in apparent sequestration of host pigments by shrimp. Consistent results were obtained also for congeneric species *T. distinctus* (Western Atlantic) and *T. spongicola* (Mediterranean). The distribution of shrimps among sponge hosts (mostly solitary individuals or heterosexual pairs) suggests that *Typton* shrimps actively prevent colonisation of their sponge by additional conspecifics, thus protecting their resource and reducing the damage to the hosts. We also demonstrate feeding on host tissues by sponge-associated shrimps of the genera *Onycocaris, Periclimenaeus*, and *Thaumastocaris* (Pontoniinae) and *Synalpheus* (Alpheidae). The parasitic mode of life appears to be widely distributed among sponge-inhabiting shrimps. However, it is possible that under some circumstances, the shrimps provide a service to the host sponge by preventing a penetration by potentially more damaging associated animals. The overall nature of interspecific shrimp-sponge relationships thus warrants further investigation.

## Introduction

Marine sponges (Porifera) are an extremely diverse group, mainly because of niche differentiation in their structurally complex habitat, which also encompasses coral reefs, mangrove islands and seagrass meadows [1]. Many sponge species have a morphological architecture of one or more large oscular openings, and a body permeated by a vast network of canals of varying diameter. This provides excellent shelter for a wide variety and abundance of small-sized symbiotic animals, including crustaceans. Some of these would-be intruders are deterred by the toxic secondary metabolites of sponges, which are considered a powerful defence mechanism [1]. Despite this, many organisms have been able to pass these biochemical barriers; up to thousands of individuals of polychaetes, amphipods, ostracods, isopods, shrimps, and ophiuroids have been found living inside individual sponges [2], [3], [4].

A wide variety of shrimps (Crustacea: Decapoda: Caridea), mainly from the families Palaemonidae (subfamily Pontoniinae), Alpheidae, and Anchistioidae have been reported as associated with sponges. Among sponge specialists, pontoniine shrimps of the genera *Periclimenaeus* Borradaile, *Typton* Costa and *Onycocaris* Nobili [5], and also alpheids of the genus *Synalpheus* Bate, are most speciose, with the last-mentioned genus providing also the first (and the only known) marine example of eusociality [6].

Although symbiotic associations with sponges may represent all types of life styles ranging from mutual benefit to parasitism [7], [8], [9], literature records discussing the nature of shrimp-sponge associations are mostly vague. Arndt [10] suggested that *Typton tortugae* McClendon, 1911 and a few *Synalpheus* spp. probably are sponge predators, and Rützler [1] classified some sponge-inhabiting alpheid shrimps as feeders on their host's tissues. An unambiguous proof of a parasitic relationship of shrimps and sponges was shown for species in the *Synalpheus gambarelloides* group, obligate inhabitants of living sponges. They were reported collecting food from the surfaces of sponge canals [11], and microscopic examination of stomach contents from collected *S. regalis* Duffy, 1996 revealed only sponge spicules and flocculent material, confirming that these shrimps feed primarily or even exclusively on sponge tissue [11]. In most other cases, however, the true nature of the shrimp–sponge relationship remains unknown. Thus, the shrimps found living inside sponges are usually referred as ‘spongobionts’, ‘commensals’, ‘inhabitants’, or ‘associates’, without specifying the actual nature of the symbiosis.

During investigation of ecology and diversity of Caribbean sponge-associated shrimps from the Belizean Barrier Reef, we collected sponges from several different habitats. Systematic review of all sponge shrimps identified within these studies will be described in a subsequent paper. In this study, we focus in detail on a relationship of two taxa: Caribbean fire sponges *Tedania* spp. (Demospongiae: Poecilosclerida) and their associated shrimp *Typton carneus* Holthuis, 1951 (Decapoda: Pontoniinae). The detailed examination of shrimp morphology, particularly an extremely developed shearing type of the claws and specific abrasion on their surface, motivated us to test the hypothesis that this shrimp may eat tissues of its host, and that such a relationship is common among sponge-associated shrimps.

To determine the feeding relation between *Typton* and its host *Tedania*, as a partial aspect of their overall symbiotic relationship, we focused on morphological adaptations and phenotypic characters of fire-sponge shrimps that may be related to their feeding ecology, and examined their stomach contents. Finally, we evaluated these relationships in several other sponge-inhabiting pontoniine shrimps from the Caribbean as well as other biogeographic regions, by examination of their morphology and diet.

## Materials and Methods

### Collecting sites

Our investigation of sponge symbionts occurred during three separate periods from 2004 to 2006 (May 14–26, 2004; October 16–22, 2005; June 6–18, 2006) in the vicinity of Tobacco Caye on the Belizean Barrier Reef, Caribbean Sea. Tobacco Caye (16°53.9′N, 88°03.7′W) is a five-acre coral island located behind the south end of a long stretch of unbroken barrier reef crest, the Columbus Reef. The island is located only 100 meters behind the reef crest, separated from it by a shallow backreef of seagrass meadow. Surrounding Tobacco Caye, between the reef crest and the mainland to the west, is a vast shallow seagrass lagoon with scattered patch reefs and mangrove islands. We investigated seagrass beds in the lagoon 100–300 m north of Tobacco Caye and 200–300 m behind the reef crest. Additionally, we collected sponges growing on mangrove roots in Tobacco Range (16°52.8′N, 88°05.7′W), a mangrove bank situated about 4 km south-west from the Tobacco Caye.

### Sponge hosts and habitats

**Figure 1:**
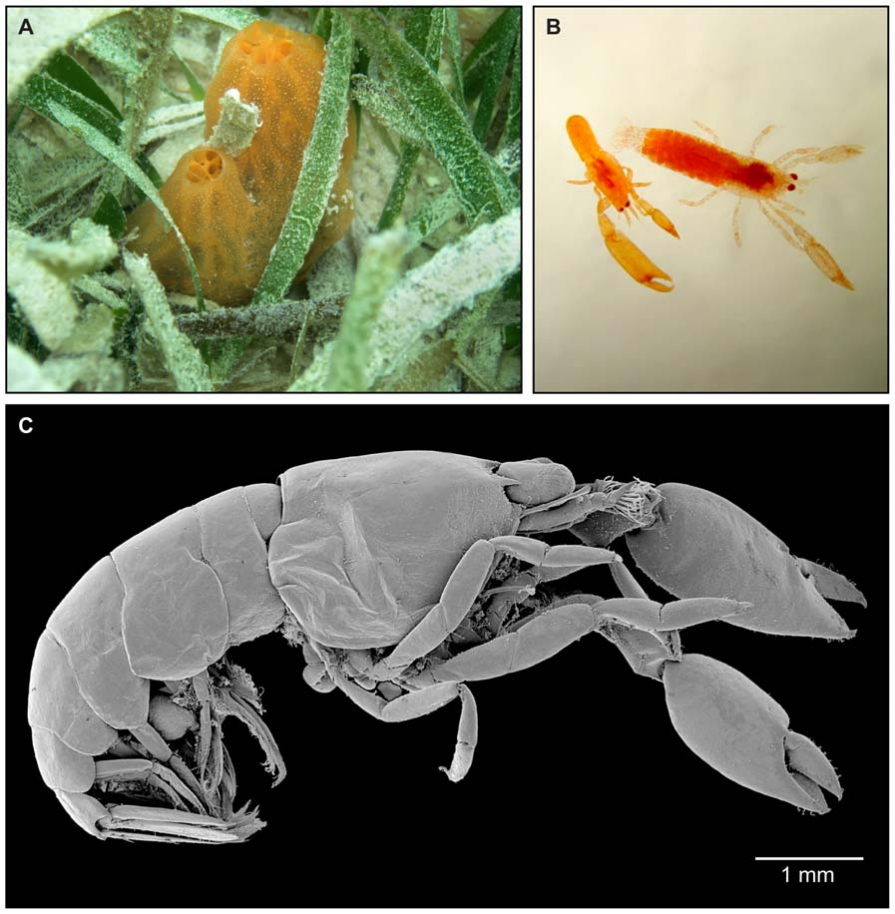
Fire sponges *Tedania klausi* (A) in the seagrass habitat of Tobacco Caye backreef, Belizean Barrier Reef, Caribbean Sea, and their obligatory associated shrimps, *Typton carneus* (B–C). A, in situ habitus of living fire sponges. B, male (left) and ovigerous female (right) pair of *T. carneus* extracted from the host sponge. C, SEM photograph of *T. carneus* showing subcylindrical smooth body with reduced and unexposed processes and spines as adaptation to life inside sponge channels, and strong, laterally flattened claws with triangular shearing fingers.

Fire sponges of the genus *Tedania* Gray usually form between 4 and 12 prominent, deep red to orange coloured ‘chimneys’, each about 15 cm high, which encrust bases and lower parts of seagrass leafs (Fig. 1A). In our study, we considered such compound, multi-oscular, sponges as individual specimens. At the top of each chimney, a single large osculum, about one centimeter in diameter, is located; this may be contracted upon disturbance. A network of internal walls separating subdermal and other internal channels from each other is visible through that opening. The sponge tissues are fine, fragile, and easily dissected, consisting of soft organic components and siliceous monaxon spicules. Fine sharp spicules of *Tedania* may irritate human skin, and toxic substance from sponge tissues cause severe dermatitis to people [12]; despite that effect, these sponges are consumed by spongivorous reef fishes. The predation pressure restricts the sponge distribution to cryptic locations on reefs; thus, they are better developed on mangrove and seagrass habitats with lower predation pressure than in reef habitats [13], [14].

*Tedania klausi* Wulff, 2006 has been reported from only two localities – Twin Cays, Belize, Caribbean Sea (type locality), and Bocas del Toro, Panama, Gulf of Mexico [7]. Our work provides an additional distributional record which is, however, only several kilometres from the type locality. The actual range needs a revision since *T. klausi* was only recently distinguished from its relative, a sympatric and widely distributed fire sponge *Tedania ignis* (Duchassaing & Michelotti, 1864) that grows predominantly on mangrove roots [7].

At Tobacco Caye, *T. klausi* mostly inhabits the backreef shallow lagoons densely overgrown by seagrass meadows dominated by turtle grass *Thalassia testudinum* (Banks & Solander ex König, 1805). In this habitat, *T. klausi* is the most common sponge species. In lesser extent, it also grows in mangrove habitat, in grass beds, and on submerged roots of the red mangrove *Rhizophora mangle* L. At the mangrove island collection site, however, the congeneric fire sponge *T. ignis* and the green sponge *Lissodendoryx* sp. were two of the most frequently observed sponge species. Although our study focused mainly on shrimps inhabiting *T. klausi*, the two above-mentioned sponges were also collected and examined, along with their associated shrimps, for comparative purposes (Table 1).

**Table 1:** Shrimp species analysed for stomach content.

Species	region	[n]	stomach content present [n]	sponge skeleton elements found	character of shrimp association to sponge host, and assumed feeding mode
**Pontoniinae**					
*Cuapetes americanus* (Kingsley)	WA	2	2	no	occasional associate, free-living
*Onycocaris spinosa* Fujino & Miyake	IWP	2	1	spongin	obligatory associate, parasitic
*Periclimenaeus caraibicus* Holthuis	WA	5	3	spicules	obligatory associate, parasitic
*Periclimenaeus rastrifer* Bruce	IWP	1	1	no	obligatory associate, parasitic (?)
*Thaumastocaris streptopus* Kemp	IWP	2	2	spicules	obligatory associate, parasitic
*Typton carneus* Holthuis	WA	5	5	spicules	obligatory associate, parasitic
*Typton distinctus* Chace	WA	4	1	spicules	obligatory associate, parasitic
*Typton spongicola* Costa	EA	2	2	spicules	obligatory associate, parasitic
**Alpheidae**					
*Synalpheus* cf. *hastilicrassus* Coutière	IWP	5	3	spongin	obligatory associate, parasitic

Soft tissues are not mentioned if sponge skeleton elements found. Abbreviations: EA, WA: Eastern and Western Atlantic (respectively); IWP: Indo-West Pacific; n: number of examined specimens.

### Sampling

Sponges were collected by snorkelling or scuba diving. Each sponge was covered by a numbered zip-locked bag which was closed immediately after detachment of the sponge from its substratum. Sponge individuals were photographed and measured as soon as possible after returning to the field base, then they were carefully dissected to pieces and all organisms found living inside were noted. Crustaceans and pieces of tissue of selected sponges were preserved in ethanol.

### Shrimps

Apart from shrimp species extracted from examined sponges—*Typton carneus*, *T. distinctus* Chace, 1972, and *Periclimenaeus caraibicus* Holthuis, 1951—we examined stomach contents and/or morphology of selected other sponge-associated shrimps. The genus *Typton* was also represented by two more lots of *T. distinctus* from Tobacco Caye (June 12 and 13, 2008, from *Tedania klausi*, coll. by F.S.), museum specimens from Nationaal Natuurhistorisch Museum – Naturalis, Leiden (RMNH) of *T. carneus* (RMNH D 23124, 51390), paratype specimens of *T. distinctus* (RMNH D 9257), all collected from Florida, and *T. spongicola* Costa, 1844 (Adriatic Sea, 1974; collection of Z.Ď.). Additional species available in the authors' collection, which lived in different sponge hosts, were examined for their morphology and stomach content: *Cuapetes americanus* (Kingsley, 1878) (Tobacco Caye, Belize, 2006), *Onycocaris spinosa* Fujino & Miyake, 1969 (Aqaba, Jordan, 2008), *Periclimenaeus rastrifer* Bruce, 1980 (Aqaba, Jordan, 2008), *Thaumastocaris streptopus* Kemp, 1922 (Nhatrang Bay, Vietnam, 2006), and *Synalpheus* cf. *hastilicrassus* Coutière, 1905 (Socotra, Western Indian Ocean, 1984).

Shrimps collected from the field were photographed alive and preserved in 70% ethanol for subsequent identification. Morphology of most taxa was studied primarily under stereomicroscopes and light microscopes, details of surface ultrastructure of *Typton carneus* were documented by scanning electron microscopy. For this purpose, shrimp individuals or their body parts were washed in 96% ethanol, dehydrated in graded acetone series, dried out by critical point drying in the BALT-TEC CPD 030, gold-coated for 5 minutes in the BAL-TEC Sputter Coater SCD 050 in argon plasma at 10^−1^ millibar vacuum, and examined with the scanning electron microscope JEOL JSM-6380 LV at 12–15 kV. Sponge skeletons and stomach contents of shrimps were examined and photographed with Canon PowerShot G9 and Olympus C5060WZ cameras attached to light microscopes.

The only biometric abbreviation used in the text, TL, refers to the total body length of a shrimp measured in the body midline from the anterior tip of the rostrum to the posterior margin of the telson.

## Results

### Shrimp presence in sponges

A total of 50 specimens of *Tedania* fire sponges, predominantly *T. klausi* from seagrass habitat, were collected during the field work from 2004 to 2006; seven specimens (containing both *T. klausi* and *T. ignis*) originated from mangrove roots. From these 50 fire sponges, 22 (44%) did not contain any associated shrimps; 17 sponges from the remaining 28 (i.e., 61%, or 34% from all samples) contained *Typton* shrimps: *T. carneus* in 15 sponges (only in *Tedania klausi*), and *T. distinctus* in two sponge hosts (one *Tedania klausi* and one *T. ignis*). Five of these *Typton* samples (four with *T. carneus* and one with *T. distinctus*), all from fire sponges from a seagrass bed, were represented by a male-female pair. One of these pairs, from an extremely large fire sponge of about 25 chimneys, was sharing the host with three additional juvenile specimens.

Our findings show a strong association between *Typton* and *Tedania* sponge hosts, which is likely underestimated by the 34% figure in this study. In June 2010 FS returned to Belize and collected twenty *T. klausi* from the same sea grass beds as previously and found that twelve sponges (60%) contained *Typton* (range: 1–8, average 2.25). As found earlier, *Tedania* typically contain only one to three shrimps, usually a male-female pair, with the majority of adult females bearing eggs. The sponge containing 8 *Typton* specimens is an atypical finding, and all of those individuals were tiny juveniles (<5 mm) or small sub-adults (5–7 mm). The size of adult specimens collected in 2006 ranged 7.3–11.0 mm, the largest ovigerous females were 8 mm of TL. Six *T. distinctus* specimens collected in 2006 and 2008 ranged 6–13 mm of TL with the ovigerous females reaching 8–13 mm.

Among other shrimps collected from the *Tedania* sponges, only *Periclimenaeus caraibicus* occurred in higher frequencies (in 9 sponge specimens, i.e., 18%). However, almost all were juvenile. On the other hand, this pontoniine species was present in all examined *Lissodendoryx* green mangrove sponges, where it occurred in groups of 2–10 specimens per one multi-oscular host. One *Lissodendoryx* sponge also hosted a single juvenile specimen of *Typton distinctus*, in addition to five *P. caraibicus* shrimps.

### Claw morphology

**Figure 2:**
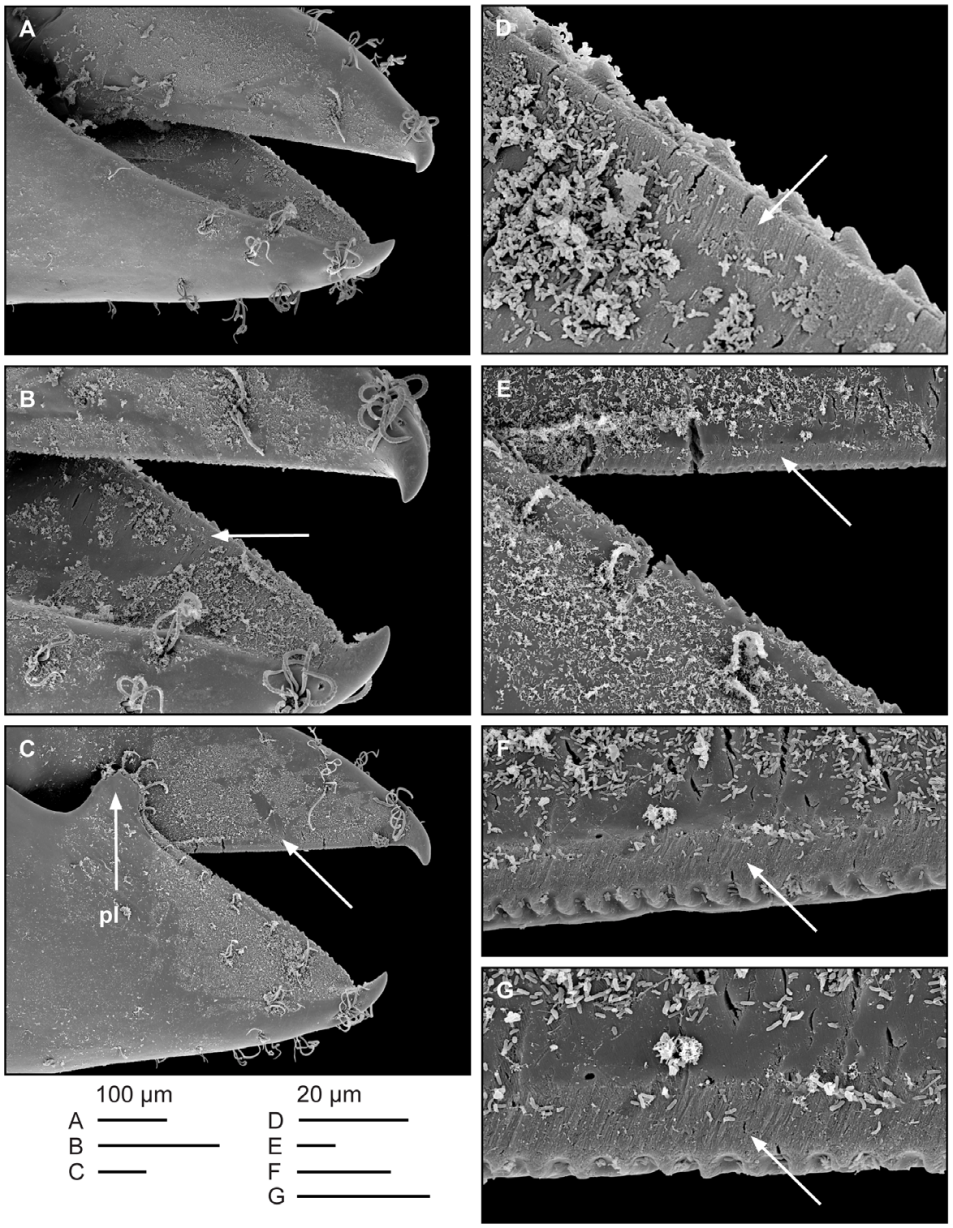
SEM details of *Typton carneus* Holthuis, 1951 female claws exhibiting traces of intensive shearing actions along the cutting edges. A–C, shearing fingers of the right (A) and left (B,C) chelae. D, a detail of the cutting edge of the right chela finger. E–G, details of cutting edges of the movable finger of the left chela. Unmarked arrows indicate traces of abrasion on cutting edges; ‘pl’ indicates the proximal lobe of the pollex.

*Typton carneus* has large, asymmetrical chelae (‘claws’) on the second pair of legs (pereiopods). In males, smaller than females, the chelae are distinctly unequal, with the larger (major) chela more developed than in adult females (Fig. 1B,C). The chelae of the second pereiopods of *T. carneus* females are different in size, but similar to each other in shape, and closely resemble the smaller (minor) chela of males. These chelae are particularly remarkable because of the morphology of the fingers and their cutting edges. Both fingers, the movable finger (dactylus) and the fixed finger (pollex), are compressed from the sides and triangular in shape – high at bases and regularly tapering to their hooked tip, similar to scissors or garden shears. The fingers are not directly opposing, but overlap each other basally when outspread. Their cutting edges are straight or regularly incurved and, when outstretched, cross each other proximally in a point; this point of overlap moves distally along the edges as the fingers close, similarly to closing scissors. The scanning electron microscopy of the chelae showed that the cutting edges exhibit narrow trails of intensive mechanical abrasion on their vertical walls (Fig. 2E–G). These trails are about 10 µm wide paths of vertical abraded grooves and bear scratches densely engraved along the cutting margins where the dactylus and pollex most intensely rub against each other.

Chelae of *Typton carneus* have the cutting edges supplied by a series of denticles (Fig. 2D–G), with larger, recurved, denticles being on the pollex (Fig. 2D). We observed this character in all examined specimens of *T. carneus* and *T. distinctus* from Belize, and also museum (RMNH) specimens of these species; such denticulation was present on both second pereiopod chelae of both sexes, with the exception of a major chela of one large male individual with only a trace of denticulation on the dactylus. A further SEM examination of *T. carneus* dactylar denticulation revealed that the apparent denticles visible in the optical microscopes are actually a series of short parallel micro-ridges or keels interspaced by narrow, deep furrows. These alternating ridges are located perpendicularly to the edge along the whole lower margin of the movable dactylus (Fig. 2E–G). The shearing profile of these ridges, visible as denticulation, keeps the cutting edge continuously sharp and functional by its abrasion during shearing actions (much like the whet-stoning of a scythe blade). In the pollex, the margin of the cutting edge in contact with dactylus is continuous, not denticulate (Fig. 2D); however, slightly laterally from this edge is a longitudinal row of triangular, backward directed denticles, spaced approx. 10 µm apart. These denticles are about twice as large and wide as profiles of the dactylar cutting micro-keels.

In contrast to *T. carneus*, the major chela in some other *Typton* species, e.g., *T. distinctus*, or the type species of the genus, *T. spongicola*, are noticeably modified in shape from the minor chela in large individuals of both sexes. In *T. distinctus*, the fingers of the major claw are strongly incurved, with the dactylus crossing the fixed finger; the sharp cutting edge is lacking on the dactylus, leaving the chela functionless for shearing actions. That, however, remains strong and powerful, and may be useful for fights. Both *T. distinctus* and *T. spongicola* have also the minor chela of the shearing type but in *T. spongicola* it lacks the cutting denticulation.

A prominent morphological feature that accompanies both the cutting and crushing types of claws in most sponge shrimps regardless of their generic affinity is a large subtriangular lobe on the proximal end of the cutting edge of the fixed finger. During the claw movement, this lobe is in continuous contact with the medial surface of the movable finger; an indistinct shallow depression may be seen on the *T. carneus* chela (Fig. 2C) as a trace of the proximal lobe of the pollex gliding on the proximo-medial surface of the dactylus.

### Mouthparts

Mandibles are mouth appendages highly adapted to cut and crush the food in the mouth before swallowing. These of examined species of *Typton* are remarkable by a reduced incisor process, which is usually responsible in shrimps for cutting food. It is slender in *T. carneus* and *T. spongicola*, with a series of minute subdistal denticles, or is deeply reduced to a low subtriangular process, as in *T. distinctus*. Also the molar process, used for crushing food particles in the mouth, is reduced in members of this genus. Instead of being widened into several strong crushing lobes, the smooth surface of the process is obliquely tapering, and ended by 2–3 slender marginal teeth.

### Stomach contents

**Figure 3:**
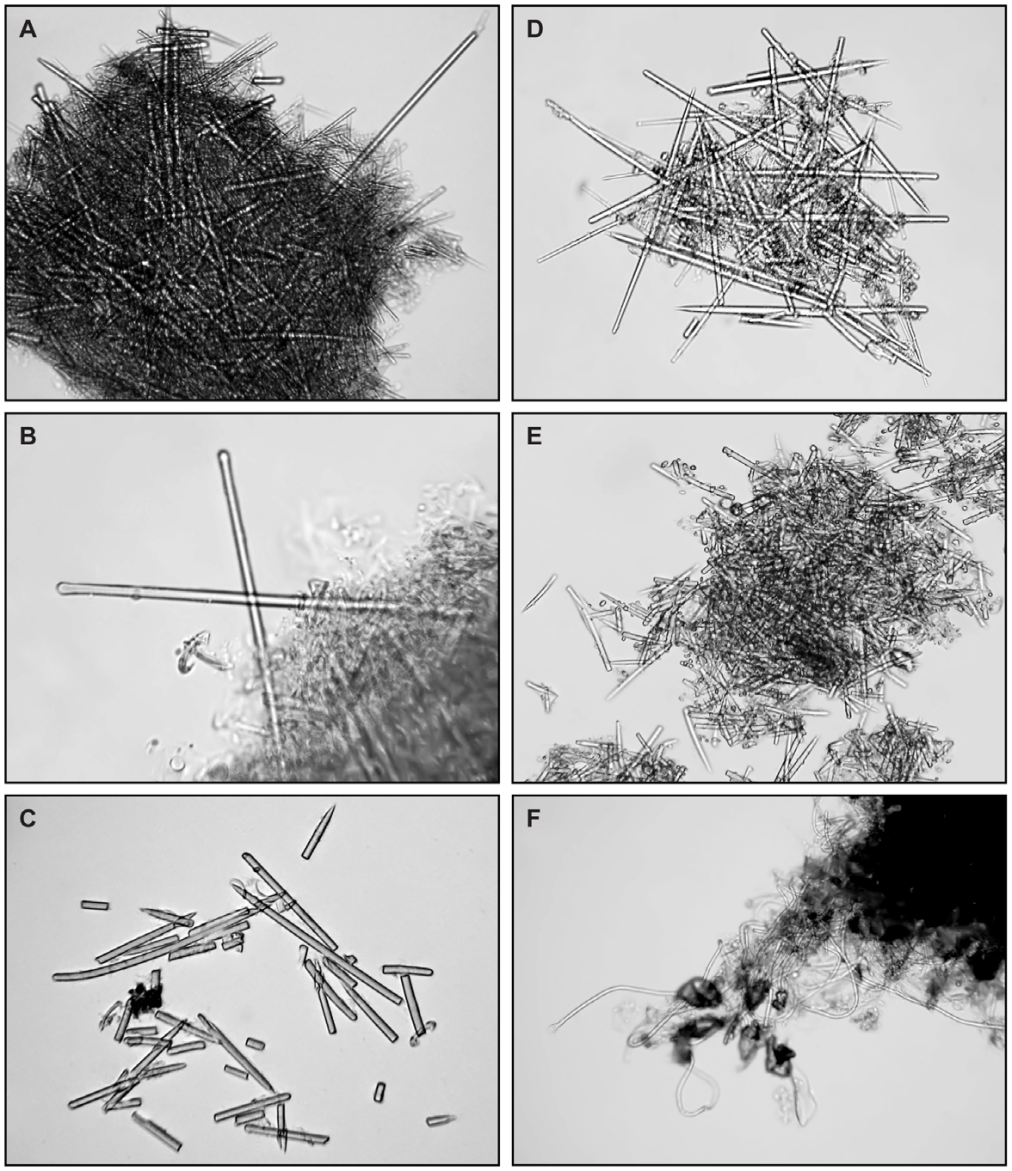
Stomach contents of spongobiotic shrimps from the Caribbean Sea (A–C), the Mediterranean Sea (D), the South China Sea (E), and the Indian Ocean (F). A, *Typton carneus* Holthuis, 1951. B, *Typton distinctus* Chace, 1972. C, *Periclimenaeus caraibicus* Holthuis, 1951. D, *Typton spongicola* Costa, 1844. E, *Thaumastocaris streptopus* Kemp, 1922. F, *Synalpheus* cf. *hastilicrassus* Coutière, 1905.

Microscopic analysis of stomach contents of dissected *T. carneus* individuals revealed food packs consisting of both a soft component and a large number of spicules, all broken into pieces (Fig. 3A). These skeletal elements were identical with the ones from the host sponge tissues. Examination of stomachs of some other sponge shrimps yielded similar results (Table 1). Sponge spicules were found also in stomachs of Caribbean specimens of *Typton distinctus* (Fig. 3B) and *Periclimenaeus caraibicus* (Fig. 3C), the Mediterranean *Typton spongicola* (Fig. 3D), and the Indo-West Pacific *Thaumastocaris streptopus* (Fig. 3E). Spongin filaments were present in food packs from *Onycocaris spinosa* and the alpheid *Synalpheus* cf. *hastilicrassus* (Fig. 3F) from the Western Indian Ocean.

### Female abdomen

The abdomen of adult females of *Typton carneus* is flattened, wide, with extended pleurae, providing a spacious marsupium for a large egg mass. Marsupia were particularly well developed in grown *T. distinctus* females, which we had available from other localities than Tobacco Caye.

### Shrimp colour and shrimp location within the host

The intensive orange-red colour of *Typton carneus* matches its sponge host background. The trend of similarity in colouration between host and shrimp was also observed in the other examined pontoniine species from Caribbean sponges: all *Periclimenaeus caraibicus* individuals inhabiting the green mangrove sponge *Lissodendoryx* spp. were of pale green colour; however, the juveniles of this species found in fire sponges were feebly pink or yellowish. None of these species was ever recorded outside of the respective sponge host, on its surface, or in the immediate area of the oscular opening. Shrimps appear to be located deeply within the sponge interior.

## Discussion

Our results strongly suggest that sponge-inhabiting shrimps are in many—possibly most—cases parasites of their hosts. The parasitic feeding habit does not necessarily mean that the damage caused by the shrimp is not balanced by other benefits provided to the host; however, we are not aware of any service that shrimps provide to the sponge. Most evidence for feeding on the host body was accumulated for Caribbean *Typton* species (*T. carneus* and *T. distinctus*); however, stomach analyses confirmed that many other taxa also feed on their host tissues (Table 1). Sponges, being one of the most evolutionary basal animal groups, have a high ability to regenerate their tissues [15], [16]; removal of internal sponge walls by spongobionts may be regenerated by the host, and therefore provide the shrimps with a continual food source. As noted by Rützler [1], endobionts of other sponges, such as alpheid shrimps, eat modest quantities of their host tissues, usually not more than can be easily replaced. Shrimps are not the only crustaceans with this feeding strategy; sponge spicules were found also in foreguts of sponge-dwelling amphipod crustaceans (e.g., [17], [18]).

The morphology of chelae of *Typton carneus* reflects the parasitic feeding habit of this species. The cutting edges of both female claws, and the minor chela of adult males, seem to be well adapted to shearing of sponge spicules, as the traces of abrasion on their surface confirm. The large subtriangular lobe situated on the proximal end of the cutting edge of the fixed finger, which leads and controls the dactylus by its movement against the opposing fixed finger, is characteristic for shrimp groups in which the claws are specialised either for shearing (as in *Typton*) or sound-producing actions (as in alpheid ‘snapping shrimps’ or the pontoniine genera *Periclimenaeus* or *Thaumastocaris* [19], [20]). Such claws are adapted to use high power and/or speed when closing, and require for their function an exact trajectory of the movement of the closing dactylus to meet opposing structures of the fixed finger.

Bruce [19] assumed that relatively large chelae of *Typton* (in comparison with free-living shrimp species) are adaptations to sponge habitat, and later ([21] p. 91), he also commented that the pollex of such chelae in *T. manningi* Bruce, 2000 acts as a shear against dactylus. Nevertheless, he did not speculate further about the function of this type of claw. Interestingly, morphologically similar chelae of *Epipontonia spongicola* Bruce, 1977, another sponge-associated pontoniid shrimp, were classified as ‘unspecialised’ in Bruce [22]. Based on our observations, we assume that they have identical function as those of *Typton*.

The denticulation of the cutting edges of *T. carneus* and *T. distinctus* claws, a feature that likely increases the shearing efficiency, is constant in both chelae of females and the minor chelae of males. Such armament was reported by Bruce [21] also for *Typton manningi*, and similar claw denticulation is present in a number of *Periclimenaeus* shrimps inhabiting sponge or ascidian hosts (e.g. [23], [24]). The claw denticulation has not been reported for the two Caribbean species examined here, and is not mentioned in their original descriptions [25], [26]. We therefore assume that this anatomical feature, apparently an adaptive one, has been either overlooked or remained uncommented by previous investigators in various other *Typton* species, and is likely to be more widespread in the genus. However, some *Typton* species, such as *T. spongicola*, clearly lack it.

The major chela in *Typton*, especially in males, is likely used in fighting with intruders. The defence of host sponges or corals by symbiotic shrimps has already been mentioned in literature (e.g. [9], [27], [28], [29], [30]). Duarte and Nalesso [4] report that *Typton gnathophylloides* Holthuis, 1951 inhabiting Brazilian sponge *Zygomycale parishii* (Bowerbank, 1875) may prevent the invasion of commensal ophiuroids, which have then to restrict themselves to smaller peripheral sponge chambers; presumably, the large claw of males has an important role in interspecific agonistic interactions. If shrimps succeed in defending their sponge hosts against intruders that may cause the sponge significantly more damage than the shrimps themselves, such service may outweigh the relatively modest damage caused by feeding on host tissues. However, we are not aware of any sponge-associated invertebrate that would have stronger impact. In the *Typton*-*Tedania* association studied by us, we did not observe associated animals that would colonise shrimp-free sponges and may have a potential to harm these hosts. This was despite the fact almost half of the investigated sponges were not colonised by any shrimp. If shrimps provided a ‘protection’ service to the sponge host, we would expect that shrimp-free sponges would be more likely colonised by other potentially harmful organisms.

Additionally, the major chela may be used for intraspecific interactions. If resident shrimps prevent adult conspecifics from settlement, or expel co-occurring juveniles after maturation, most suitable but small-sized sponges should be typically inhabited by a solitary individual or a pair [29]. This is consistent with our observations. We assume that the shrimps usually enter a suitable sponge during their postlarval stage, at the end of the planktonic phase. There they mature, and live permanently within the interior of the sponge, defending it from both conspecific and heterospecific shrimps as well as other intruders. Thiel & Baeza [29] showed that strong weaponry (enlarged claws) of symbiotic crustaceans has developed as an adaptation to intraspecific, more likely than interspecific, relations. Simple and less complex hosts may be easily defend by heterosexual pairs of symbionts from conspecifics to ensure limited utilization of host sources. This territoriality might be a result of evolution towards lower impact on the sponge, as is often the case for endoparasites intimately associated with their host.

The mouth apparatus of the examined species also supports specialisation on feeding on sponge tissues. The mandibles of various *Typton* species are remarkable for a reduction of their incisor process (responsible for cutting food). The extent of this reduction differs [21], [25], [26], [31]: from a slender process with minute distal denticulation, as in *T. carneus*, *T. manningi*, or *T. tortugae*, to a substantially more reduced, low subtriangular process as in *T. distinctus* or *T. dimorphus* Bruce, 1986, or even complete disappearance of this structure, e.g., in *T. gnathophylloides*. The molar process, responsible for crushing food in the mouth, is also rather slender in *Typton* species, and bears slender sharp teeth (suitable to hold and crush the sponge spicules) instead of obtuse robust knobs as usual in majority of free-living caridean shrimps. A similar mandibular arrangement is known also in some sponge-dwelling amphipods, e.g., *Paramphithoe hystrix* (Ross, 1935), a micropredator on North-West Atlantic sponges. Its incisor process is oblique and toothed, allowing the amphipod to shear through the spongin fibers, and the molar process is modified to handle the sponge spicules [17].

Reductions of the mandibles noted above for *Typton* occur also in pontoniine shrimps of some other genera. The reduced incisor process and somewhat tapering, sharply toothed, molar process were reported for example in *Epipontonia spongicola*, *Onycocaridella antokha* Marin, 2007, *Onycocaris amakusensis* Fujino & Miyake, 1968, *Periclimenaeus caraibicus*, *P. pearsei* (Schmitt, 1924), *P. schmitti* (Holthuis, 1951), *Poripontonia dux* Fransen, 2003, *Typtonychus crassimanus* Bruce, 1996 [22], [24], [25], [32], [33], [34], and most of their congeners.

Apart from securing food supply, spongobiotic shrimps also face a problem of limited supply of suitable hosts. The widespread presence of a single pair of shrimps in a host suggests that settlement of additional individuals is actively prevented by the original inhabitants. In such case, females invest into production of a greater number of eggs, increasing the chances that their offspring successfully colonise new hosts. Indeed, we observed that the abdomen of fully grown adult females of *T. carneus* has a spacious marsupium. In comparison with other similarly-sized pontoniine shrimps, this chamber with long slender pleopods is adapted to accommodate a large number of eggs. *Typton* females also have extremely enlarged ovaries, which unusually reach from cephalothorax to anterior abdomen, thus ensuring an enlarged volume for produced oocytes; some other spongobiotic pontoniine genera also show this feature, although in a lesser extent [19]. Such characteristics show that these spongobiotic shrimps have a reproductive mode typical for r-strategists, which is clearly another adaptation to obligatory symbiotic relationship with their sponge hosts. In distinct cases, however, sponge-shrimps may undergo even direct development and parental care, as shown by Duffy [6] for eusocial *Synalpheus* shrimps.

Although the key factor causing reduction of parasite virulence and evolution towards mutualistic relationship is a vertical transmission of parasites from the parent host to its progeny, reduced virulence may develop also without such transmission [35]. The presence of planktonic larval phase in the life cycle of *Typton* shrimps as well as their hosts does not allow for vertical transmission. In that case, the theory predicts increased importance of higher fecundity of the symbiotic organisms, as observed in various shrimps (including *Typton*) that live in small internal cavities of various invertebrate hosts ([19] Z.Ď., pers. obs.).

The intensive orange to cherry-red colour of adult *T. carneus* matches well its sponge background. Subadult *Typton* specimens are of a less intense red colour, and the tiny juveniles are pale red to light pink in colour. Although it could serve as a cryptic colouration, shrimps seem to always remain inside the sponge, in the largest and deepest internal chambers ([4] Z.Ď., pers. obs.), where such adaptation is not needed. The colouration may rather be a result of sequestration of pigments from consumed sponge tissues; such use of host pigments was already shown for other symbiotic shrimps that are obligatory associates of sea urchins [36], [37]. The hypothesis that spongobiotic shrimp incorporate pigments from the host is also supported by our observation of colouration of *Periclimenaeus caraibicus* associated with green *Lissodendoryx* sponges. Both adult and juvenile individuals were pale greyish-green; however, juveniles found in the red fire sponges were feebly pink to yellowish. Additionally, we observed one pair of this species in a different host, the purple sponge *Hyrtios violaceus* (Duchassaing & Michelotti, 1864) in the seagrass habitat. This sponge is coloured by phycobilins originating from symbiotic filamentous algae [38], easily released to water upon mechanical damage. *Periclimenaeus caraibicus* specimens from a purple *Hyrtios* host were light purple. Thus, colouration of other common Caribbean spongobiotic shrimp species, if related to the colouration of the host, is probably achieved by feeding of host sponge tissues that contain the respective pigments.

Although we directly confirmed the parasitic feeding mode for only a limited number of shrimp species (Table 1), we assume that the same applies to many spongobiotic shrimps of similar morphology. Together with other species of *Typton* (e.g. [21], [25], [26], [31], [39]), analogous shear-like claws are also found in some other pontoniine shrimps. Fransen [34] and Bruce [40] list 18 sponge-associated pontoniine genera, with a possible additional one, *Typtonides* Bruce, 2010 [41]. Among these, such claws were described at least for selected species of the genera *Periclimenaeus* Borradaile, *Poripontonia* Fransen, *Typtonychus* Bruce, and *Anisomenaeus* Bruce (e.g. [24], [33], [34]). For *Anisomenaeus* and *Typton*, the shearing type of the minor chela is an important diagnostic character [40], [42], [43]. The minor chela of most pontoniines of the genera *Epipontonia* [22], [44], [45], *Onycocaris* Nobili and *Onycocaridella* Bruce [40], [42], [46] also show a possible shearing shape and function, and our results prove feeding on sponge for *Onycocaris spinosa*. Similarly, shrimps of the genus *Discias* (fam. Disciadidae), considered to be sponge commensals, have also shearing chelae of a highly specific shape [47].

Shearing type of claws apparently evolved several times independently as an adaptation to a spongobiotic mode of life. The differences in claw morphology among sponge-associated genera may also depend on the character of different sponge hosts, nature of their skeleton elements, and structure of internal habitat. Many demosponges have tough, dense, or elastic skeletons, which are difficult to shear; despite this, they are also inhabited by shrimps which probably use other mechanisms to obtain food particles. For example, a tough Indo-West Pacific sponge of the genus *Ircinia* Nardo was reported as host of the shrimps *Typton manningi* and *Periclimenoides odontodactylus* (Fujino & Miyake, 1968) [21], [48]. The claws of the latter species are not of a shearing type; these shrimps probably use the smaller claws on the first pair of pereiopods to collect food items.

In conclusion, a wide group of sponge-inhabiting pontoniine shrimps display similarities in at least some of the following features: (1) a complex of morphological adaptations to shear and crush sponge tissues, including spicules, (2) a settlement in deep sponge chambers, (3) limited access to food sources other than host tissues, (4) assumed intraspecific aggressive behaviour in some species resulting in exclusive presence of a single individual or a heterosexual pair in a host specimen, (5) r-strategy of reproduction, and, (6) possible incorporation of sponge pigments to shrimps bodies.

These facts suggest that most or all of these shrimps eat their host tissues, and, thus, their feeding relation to their hosts should be considered parasitic. In at least some of these cases, there does not seem to be any substantial benefit provided by the shrimp to the host. Because of (i) shrimp small size (usually up to 15 mm TL), (ii) a limited number (usually single specimen or monogamous pair) in any given sponge, and (iii) the ability of the sponge to regenerate damaged tissues, it is likely that the feeding actions of the shrimp on the sponge do not have severe consequences. This might be a consequence of an evolution to low virulence of the parasites; in particular, we presume that intraspecific aggressiveness may have evolved under the pressure for sustainable use of the host. On the other hand, if the shrimps show aggressive behaviour towards other potentially harmful species colonising the sponge, the benefits for the hosts may be higher than losses caused by the shrimp feeding.

The exact nature of many interspecific interactions remains unclear, with some evidence suggesting mutualism, and other parasitism, pointing to the same pair of interacting species [49]. Associations that are harmful or neutral in one ecological setting may become mutually beneficial in another (see [50]). In particular, a distinction between mutualism and parasitism may be very subtle [51]. With advancing knowledge, the same sponge-symbiont relationship may have been considered harmless, parasitic, and, finally, mutualistic [52]. On the other hand, negative costs of the symbiosis may often be hard to detect, possibly even completely hidden due to host's phenotypic plasticity [53]; this is especially true for sponge hosts that are consumed from within.

Parasitic nature of feeding ecology of sponge-inhabiting shrimps thus represents only one aspect of complex shrimp–sponge relationships. As discussed above, the costs and benefits of such association may be context-dependent. A wide range of relations, including parasitism as well as mutualism, was recently shown also for shrimps associated with some other hosts, e.g., oculinid corals [54], or sea anemones [55], [56].

The phenomenon of feeding parasitism is apparently more widely distributed among spongobiotic pontoniine and alpheid shrimps than previously considered. We suggest that similar parasitic relations will be found in many cnidarian-, mollusk- or echinoderm-associated shrimps as well as other cases of symbioses with sessile invertebrate hosts on tropical reefs.
